# Optimization and In Vitro Digestion of a Guava (*Psidium guajava*), Mamey (*Pouteria sapota*) and Stevia (*Stevia rebaudiana*) Functional Beverage

**DOI:** 10.3390/foods13010142

**Published:** 2023-12-30

**Authors:** Beatriz Haydee Belmonte-Herrera, J. Abraham Domínguez-Avila, J. Fernando Ayala-Zavala, Martín Valenzuela-Melendres, Orlando Tortoledo-Ortiz, Gustavo A. González-Aguilar

**Affiliations:** 1 Centro de Investigación en Alimentación y Desarrollo A. C., Carretera Gustavo Enrique Astiazarán Rosas No. 46, Col. La Victoria, Hermosillo 83304, Mexico; belmonteh.beatriz@gmail.com (B.H.B.-H.); jayala@ciad.mx (J.F.A.-Z.); martin@ciad.mx (M.V.-M.); otortoledo@ciad.mx (O.T.-O.); 2 CONAHCYT-Centro de Investigación en Alimentación y Desarrollo A. C., Carretera Gustavo Enrique Astiazarán Rosas No. 46, Col. La Victoria, Hermosillo 83304, Mexico; abrahamdominguez9@gmail.com

**Keywords:** mixture design, D-optimal design, phenolic compounds, carotenoids, in vitro digestion, functional beverage

## Abstract

Guava and mamey are phenolic- and carotenoid-rich fruits with potential health benefits, but are minimally used as ingredients in functional beverages. The objectives of the present work are to optimize the content of guava and mamey pulps and a stevia solution in the formulation of a functional beverage with high content of bioactive compounds and sensory acceptability using a mixture design analysis, and to analyze its composition after in vitro digestion. The optimized formulation (17.77 and 19.23 g of guava and mamey pulps, respectively; 1% stevia solution) yielded a beverage with 418.21 mg gallic acid equivalents (GAE)/100 mL and 0.20 mg β-carotene/100 mL, and an antioxidant capacity of 213.58, 78.90 and 234.03 mg Trolox equivalents (TE)/100 mL using three methodologies. The mathematical model developed was significant (*p* < 0.05), according to R^2^ values between 0.70 and 0.75. α- and β-carotene were quantified during the oral phase of in vitro digestion. Gallic, *p*-coumaric, ferulic and chlorogenic acids were also identified. The beverage had a general acceptability of 6.72. We conclude that the mathematical model developed was a good predictor of the experimental data and that the optimized beverage contained high bioactive concentrations (phenolics and carotenoids) and was well-accepted by potential consumers.

## 1. Introduction

Regular consumption of carbonated or non-carbonated sugar-rich beverages is associated with negative health effects, including obesity, type 2 diabetes, cardiovascular diseases and hypertension [1,2,3]. Some of the underlying mechanisms for this association include incomplete compensation for liquid energy, adverse glycemic modifications and increased hepatic metabolism of fructose, all of which promote de novo lipogenesis, the production of uric acid and the accumulation of visceral and ectopic fat deposits [4,5].

Some alternatives aimed at mitigating this high consumption include increased taxation on sugar-rich beverages, which has been implemented in more than 73 countries worldwide. This has been described as a triple win, since it can improve population health, generate revenue, and has the potential to reduce long-term associated healthcare costs and productivity losses [6]. On the other hand, taking advantage of the growing demand for nutritious foods with biological potential, such as antioxidant, anti-inflammatory and anti-proliferative activities, and which also have innovative tastes and flavors, the development of beverages based on exotic fruits has been proposed [7,8].

Combining two or more fruits is a strategy used by various industries to create new beverages that can accomplish several goals, such as improving their color and consistency, adding nutritional value, innovating tastes and flavors, reducing costs by adding less expensive fruits and keeping up with market trends [7,9,10]. Guava (*Psidium guajava* L.) has been considered an exotic “super fruit” due to its high content of phenolic compounds and other antioxidant substances. Several phenolic acids are found in its pulp, including gallic acid, hydroxybenzoic acid, vanillic acid, caffeic acid, syringic acid, *p*-coumaric acid, ferulic acid and synapic acid [11], in addition to other phytochemicals [12]. Mamey fruit (*Pouteria sapota*) is also considered an exotic fruit. It has a particular orange-to-red-colored pulp, due to the presence of carotenoids with *kappa* end groups. These molecules are very rare in nature, but they can be found on both the nonpolar and polar fractions of extracts from this fruit. Some specific molecules identified in it include allene carotenoids like neoxanthin, (9′Z)-neoxanthin and capsoneoxanthin [13,14]. Both fruits are harvested in Mexico, although their market is limited; thus, proposing their incorporation into new products could boost their commercial reach, while also offering the consumer a bioactive-rich product. Using non-caloric sweeteners like leaves of green stevia (*Stevia rebaudiana*) in a functional beverage is an alternative to replace added sugars [15], thereby contributing to the development of healthier alternatives [16].

In the present study, two exotic fruits (guava and mamey) were used together with a stevia solution as a non-caloric sweetener; their ratios were optimized using a mixture design, with the goal of producing a beverage of high bioactive compound content and sensory acceptability. Our results can be a good entry point for the acceptance of these exotic fruits as part of a novel formulation, which can add nutritional and sensory value to the fruit beverage, while providing a high-quality product to the consumer.

## 2. Materials and Methods

### 2.1. Standards and Reagents

All reagents used were purchased from Sigma-Aldrich (St. Louis, MO, USA), specifically, Fast Blue BB Salt hemi (zinc chloride) salt (FBBB), 2,2′-azino-bis-(3(ethylbenzothiazoline-6sulfonic acid) (ABTS), 2,2-diphenyl-1-picrylhydrazyl (DPPH), Trolox (6-hydroxy-2,3,7,8 tetramethylchroman-2-carboxylic acid), TPTZ (2,4,6-tripyridyl-s-triazine), ethanol, hexane, acetone, butylated hydroxytoluene (BHT), NaOH, HCl, acetate, ethyl ether, formic acid, methanol, *tert*-butyl methyl ether and ammonium acetate. Phenolic and carotenoids standards of gallic acid, gallocatechin gallate, *p*-coumaric acid, quercetin 3-β-d-glucoside, ferulic acid, cinnamic acid, chlorogenic acid, vanillic acid, catechin, cryptoxanthin, α-carotene, β-carotene, retinol, lutein and zeaxanthin were of HPLC-grade. In vitro digestion reagents used were pancreatin (from porcine pancreas), α-amylase (from porcine pancreas), pepsin (from porcine gastric mucosa), bile salts, KCl, KH_2_PO_4_, NaHCO_3_, NaCl, MgCl_2_·6H_2_O, (NH_4_)_2_CO_3_ and CaCl_2_·2H_2_O.

### 2.2. Plant Material

Frozen mamey pulp (−20 °C) from commercially ripe fruits was purchased from a local market in the city of Hermosillo, Mexico. Although fresh mamey fruit is sometimes sold in this region, it is seasonal, it is only found in limited amounts and only in some stores; thus, its availability is low during most of the year and in most locations. This made it necessary to acquire minimally processed frozen pulp, which was kept frozen under dry and dark conditions until processing (within 72 h). It should therefore be noticed that some minimal processing had occurred during peeling and freezing by the distributor previous to when it was used in the laboratory, and some variation could thus be expected if similar experiments were to be repeated using fresh mamey. Fresh commercially ripe guava fruits were also purchased in a local market in the city of Hermosillo, Mexico; they were transported to the laboratory and hand washed under running tap water to eliminate any surface contaminants. Seeds were manually removed from the pulp and discarded; the obtained pulp was immediately frozen at −20 °C and stored until use. Dry *Stevia rebaudiana* leaf powder was bought from a local supermarket in Hermosillo, Mexico.

### 2.3. Stevia Solutions

Stevia leaves were added to 1000 mL of boiled (100 °C) purified water to prepare stevia solutions (1.00, 1.25, 1.37 and 1.50%) [7]. The mixture was left to infuse for 30 min, and then filtered with Whatman^®^ (Maidstone, UK) No. 1 filter paper.

### 2.4. Beverage Preparation

Guava and mamey pulps were blended for 2 min in an immersion mixer (NutriBullet, Los Angeles, CA, USA, 600 W) with 100 mL of stevia solution. Purified water was then added to reach 200 mL of total volume. The three matrices were at room temperature during preparation (24 °C).

#### 2.4.1. Experimental Design, Data Analysis and Verification of the Model

Actual ingredient proportion for each experimental run and total number of experimental runs were determined. The effective ratios obtained here form the fundamental basis for the D-optimal mixture design model that derived the optimal ratio of guava/mamey/stevia.

#### 2.4.2. Constrained Formulation

The amount of each ingredient was imposed with lower and upper bounds established according to the literature, where the sensory and/or bioactive composition of each ingredient was considered. Specifically, the amount of guava pulp was set according to its sensory attributes on a beverage; the established range was between 12.22 and 17.77 g of pulp, based on the work of [17], with some modifications. The amount of mamey pulp was set from 18.73 to 24.78 g according to [18], in order to match the concentration of sapotexanthin (845 μg capsanthin) and cryptocapsin (1.21–1.51 mg capsanthin) reported by other authors in a similar beverage. Stevia leaves solution was imposed based on preliminary assays according to its sweetness.

#### 2.4.3. D-Optimal Mixture Design and Analysis

A D-optimal mixture design was used to optimize the proportions of guava pulp (X_1_), mamey pulp (X_2_) and stevia solution (X_3_). The range and central point value of all three variables are shown in Table 1, according to a fixed range that was established by the researchers based on the previous literature [17,18]. This table shows the 16-point matrix that was predicted by the software, which served to determine which ingredient combination resulted in the best bioactive concentration and antioxidant capacity. It should be noted that, as part of the optimization, the software considers identical runs, such as 15 and 16, in order to verify the reproducibility of the results. Thus, these are expected to have statistically similar values, which serve to validate experimental reproducibility.

The experimental design, analysis of the results and prediction of the responses were carried out using Design–Expert software Version 13.0 (Stat-Ease Inc., Minneapolis, MN, USA). The mathematical models were subjected to an analysis of variance (ANOVA) and a regression analysis. The significant contribution of each coefficient was determined by the *p*-value of the F-test (*p* < 0.05). The simultaneous optimization of the response variables was based on the overall desirability function.

The different ratios were numerically optimized for maximum yield of bioactive compounds and antioxidant capacities, set as the dependent variables DPPH (Y_1_), TEAC (Y_2_) FRAP (Y_3_), TPC (Y_4_) and TC (Y_5_), based on the regression analysis of the independent variables. Finally, the predicted values were compared with the experimental value to determine the validity of the model.

### 2.5. Methanolic Extracts

A methanolic extract from fresh raw materials was prepared to quantify their antioxidant capacity and total phenolic content (TPC), according to the methodology of [19], with some modifications. Pulps were blended separately for 1 min in an immersion mixer, and then, 10 g of guava and mamey pulps were weighed, as well as 1 g of powdered stevia leaves. Subsequently, 20 mL of methanol/water 80:20 (*v*/*v*) was added to each sample (guava pulp, mamey pulp and stevia leaves), which was then sonicated in an ice bath for 30 min (Bransonic, model 2510R-DTH, Danbury, CT, USA) and centrifuged at 18,000× *g* for 15 min at 4 °C. The obtained supernatant was then filtered through Whatman No. 1 paper and recovered in a 50 mL volumetric flask. The pellets were washed twice with 10 mL of the 80% methanol solution, and the sonication and centrifugation processes were repeated as previously described. Finally, volume was adjusted with 80% methanol up to 50 mL, and stored at −35 °C. The obtained extracts were later used to quantify TPC and antioxidant capacity by DPPH, TEAC and FRAP.

### 2.6. Total Phenolic Content (TPC) by Fast Blue BB (FBBB)

For the FBBB methodology, 1 mL of beverage was mixed with 100 µL FBBB 0.1% solution; it was vortexed for 1 min and 100 µL of 5% NaOH was then added. The solution was allowed to react for 90 min at room temperature under dark conditions. Then, 200 µL were pipetted into a microplate well and read at 420 nm in a microplate reader (FLUOstar Omega, BMG Labtech, Offenburg, Germany). Experiments were performed in triplicate, and a standard curve of gallic acid was used to determine the concentration of TPC. Data were expressed as mean ± standard deviation of mg gallic acid equivalents (GAE)/100 mL [20].

### 2.7. DPPH^●^ Radical Scavenging Activity

A DPPH solution was prepared by dissolving the radical in pure methanol and adjusting its absorbance (515 nm) to 0.70 ± 0.02. After that, 20 μL of beverage was pipetted into a microplate well, and 280 μL of the DPPH radical was then added. It was incubated under dark conditions for 30 min. The absorbance was read at 515 nm in a microplate reader (FLUOstar Omega). A blank was prepared by adding distilled water instead of the beverage, and serial dilutions of Trolox were used as standards; these were read under the same conditions [21,22,23]. Experiments were performed in triplicate, and results are expressed as mean ± standard deviation of mg of Trolox equivalents (TE)/100 mL.

### 2.8. Trolox Equivalent Antioxidant Capacity (TEAC) Assay

The ABTS^●+^ stock solution was prepared from 7 mM ABTS and 2.45 mM potassium persulfate, mixed at a volume ratio of 1:1, and then incubated under dark conditions for 16 h at room temperature. The ABTS^●+^ working solution was prepared by diluting the stock solution with ethanol to an absorbance of 0.70 ± 0.02 at 734 nm. The sample (5 μL) was mixed with 245 μL of ABTS^●^+ working solution and, after 5 min of incubation at room temperature, the absorbance of the reaction mixture was read at 734 nm [22,23,24]. The percentage of absorbance inhibition at 734 nm was calculated, using distilled water as blank and Trolox as standard. Experiments were performed in triplicate, and results are expressed as mean ± standard deviation of mg TE/100 mL.

### 2.9. Ferric Reducing Antioxidant Power (FRAP) Assay

The FRAP solution consisted of a mixture of 5 mL of acetate buffer (300 mM, pH 3.6), 500 μL of 2,4,6-tri-(2-pyridyl)-S-triazine (TPTZ, 10 mM in 40 mM HCl) and 500 μL of FeCl_3_·6H_2_O (20 mM). Then, 20 μL of the sample was pipetted into a microplate well, and 280 μL of the FRAP reagent was added. It was incubated for 30 min in the dark, and its increase in absorbance was read at 595 nm using a spectrophotometer (FLUOstar Omega) [22,23,25]. The final absorbance of each sample was compared to those obtained from a Trolox standard curve. Experiments were performed in triplicate, and results are expressed as mean ± standard deviation of mg TE/100 mL.

### 2.10. Total Carotenoids (TC)

Ten mL of the sample was mixed with 5 mL of a solution of acetone/BHT 0.05% (*v*/*w*), 5 mL of 95% ethanol and 10 mL of hexane. This mixture was stirred on an orbital magnetic shaker for 15 min; 3 mL of distilled water was then added and stirred for an additional 5 min. Samples were left at room temperature under dark conditions for 5 min to allow phase separation. The hexane supernatant was recovered, and its absorbance was measured at 450 nm in a 1 cm path length quartz cuvette, blanked with hexane. The data were used to calculate total carotenoid content, according to Equation (1):

(1) $$TC=(A\times V\times 10^{4})÷\varepsilon \times m$$

where TC is total carotenoid concentration; A is absorbance; V is the recovered volume of hexane from the insoluble fraction; 10^4^ is the conversion constant from g to μg; ε is the molar extinction coefficient; and m is the sample’s mass in g [26].

Experiments were performed in triplicate, and results are expressed as mean ± standard deviation of mg of β-carotene/100 mL (for beverages) or μg of β-carotene/g (for fruits).

### 2.11. Sensory Analysis

Approval for the study was obtained from the Institutional Ethics Committee by the Research Center for Food and Development (acronym in Spanish, CIAD), approval number CEI-020-3/2022. Prior to participation in the sensory analysis, each volunteer was comprehensively briefed about the nature of this study. This included detailed information about the beverage samples, the purpose and potential use of the study results and the voluntary basis of their participation. Volunteers were explicitly informed that they could withdraw at any point without repercussion. Following this briefing, oral consent was obtained in the presence of at least two members of the research team, serving as witnesses. This procedure is in alignment with our Institutional Ethics Committee’s guidelines, which are based on the National Guide for the Integration and Operation of Research Ethics Committees, the General Health Law in Health Research and the ethical principles outlined in the Declaration of Helsinki.

One hundred and ten untrained consumers participated in the sensory evaluation (male and female, from 19 to 39 years old; students, professors, research scientists and administrative staff of CIAD and the University of Sonora in Hermosillo, Mexico). Three beverages were considered for this evaluation, the optimized beverage and two runs from the mixture design. The optimized formulation had 17.77 and 19.23 g of guava and mamey pulps, respectively. Mixture 1 had the lowest amount of mamey pulp and highest of guava pulp; mixture 15 had the lowest amount of guava pulp and highest mamey pulp. Each beverage was coded with random three-digit numbers when consumers evaluated them. They were asked to rate the beverages’ color, smell, flavor, viscosity and general acceptability using a 10 cm linear 7-point hedonic scale, with anchors of “dislike a lot” on the left and “like a lot” on the right. They were also asked to state their purchase intention, and a blank space was provided if they wanted to include any comments. Participants were served 30 mL of each sample, in addition to 200 mL of water and unsalted cookies to cleanse their palate between samples [27,28].

### 2.12. Phenolic Profile

In order to identify and quantify free and chemically bonded PCs, a methanol extraction was performed, where free PCs were identified [29]. After this, sequential alkaline and acid hydrolyses were performed. For alkaline hydrolysis, 10 mL of degassed 2 M NaOH was added to 0.5 mg from the methanol extraction pellet. Oxygen was displaced with nitrogen for 15 s. Hydrolysis was performed at room temperature with constant stirring of 100 rpm, under dark conditions for 4 h. After that, pH was adjusted to 1.5–2.0 with 6 M HCl. For acid hydrolysis, 5 mL of HCl (37%) was added to 0.5 mg from the methanol extraction pellet. Hydrolysis was performed in a water bath at 85 °C for 3 h and constant stirring of 100 rpm. At the end of this hydrolysis, the pH of the solution was adjusted to 7.0 with NaOH 2 M [30].

After both hydrolyses, a liquid–liquid extraction was performed with ethyl acetate/ethyl ether (50:50; *v*/*v*). Afterwards, 15 mL of this solution was added to the hydrolyzed mixture and vigorously vortexed for 15 s; the tube was opened slightly and briefly to release gas, closed again and vortexed for another 15 s. It was then allowed to separate into two phases, and the supernatant was recovered in a rotary evaporator flask. Another 15 mL of acetate/ethyl ether (50:50; *v*/*v*) was added and the previous steps were repeated once more. After that, the 30 mL recovered were dried in a rotary evaporator at 45 °C, resuspended in 5 mL of 80% methanol, filtered with a 25 μm filter and analyzed as follows.

The identification and quantification of the phenolic profile present in the optimized beverage was carried out in a UHPLC apparatus (Acquity, Waters, Milford, MA, USA) equipped with a diode array detector, as previously reported [19]. Separation was performed on a BEH C18 column (1.7 μm, 3.0 × 100 mm, Waters) under temperature-controlled conditions (60 °C). Solvents were water with 0.5% formic acid (A) and 100% methanol (B), which were used under the following gradient: 0–0.25 min 20% of B (flow 0.4 mL/min); 5 min 20% A (0.2 mL/min); 12 min 45% B (0.180 mL/min); 25 min 100% B (0.1 mL/min); 16 min (40% B, 0.2 mL/min); and 30 min 20% B (0.4 mL/min). The eluted compounds were identified by comparing their retention times and UV/Vis and absorption spectra with their respective commercial standards and quantified using standard curves prepared with the same standards. Results were expressed as μg/mL of the sample.

### 2.13. Carotenoid Profile

For carotenoid identification and quantification, an extraction was first carried out. 500 μL of beverage were vortexed with 1.5 mL of hexane and centrifuged at 18,000× *g*, for 10 min at 4 °C. The supernatant was recovered, and the process was repeated twice. Samples were evaporated to dryness using nitrogen, and extracted solids were reconstituted in 200 μL of ethanol. Carotenoids were analyzed in the extracted samples using a reverse phase gradient HPLC method [31]. The HPLC system was an Agilent Technologies 1220 Infinity apparatus, a C30 carotenoid column (3 µm, 150 × 3.0 mm, YMC, Wilmington, NC, USA) and an Agilent Technologies 1260 photodiode array detector. Mobile phases were methanol/*tert*-butyl methyl ether/water (85:12:3, *v*/*v*/*v*, with 1.5% ammonium acetate in water) as solvent A, and methanol/*tert*-butyl methyl ether/water (8:90:2, *v*/*v*/*v*, with 1% ammonium acetate in water) as solvent B. Absorbances were read at 430, 450 and 471 nm. Identification of eluted compounds was performed by comparing their retention times and absorption spectra with their respective commercial standards and were quantified using standard curves prepared with the same standards (Sigma-Aldrich, St. Louis, MO, USA). Results were expressed as μg/100 mL of sample.

### 2.14. In Vitro Digestion

A three-stage in vitro digestion was performed. Simulated salivary fluids (SSF), simulated gastric fluids (SGF) and simulated intestinal fluids (SIF) were prepared, which contained the following salts (in mM, for SSF, SGF and SIF, respectively), KCl (15.1, 6.9 and 6.8), KH_2_PO_4_ (3.7, 0.9 and 0.8), NaHCO_3_ (13.6, 25, 85), NaCl (0.00, 47.2, 38.4), MgCl_2_·6H_2_O (0.15, 0.12, 0.33), (NH_4_)_2_CO_3_ (0.06, 0.50, 0.00), HCl (1.1, 15.6, 8.4) and CaCl_2_·2H_2_O (1.5, 0.15, 0.60). SSF was adjusted to pH 7.0, SGF to pH 3.0 and SIF to pH 7.0 [32].

**Figure 1:**
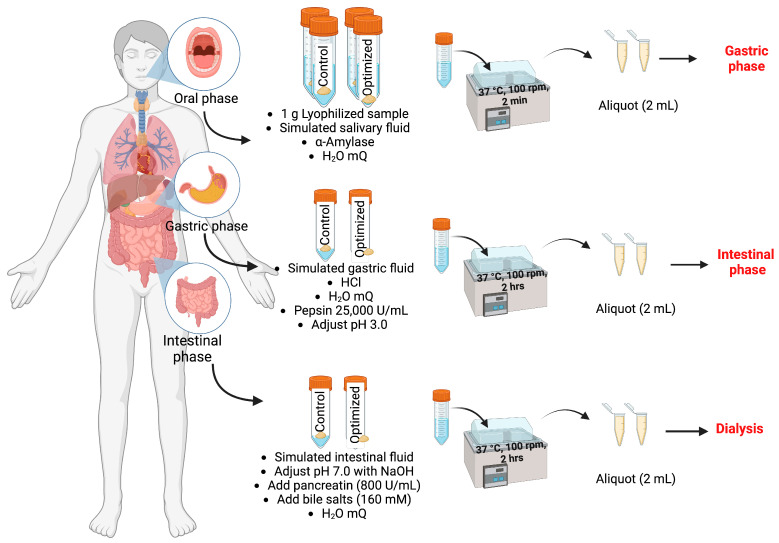
General description of in vitro digestion model.

One gram of lyophilized beverage sample was resuspended in 8.5 mL of SSF at 37 °C, to which 0.5 mL of α-amylase from porcine pancreas (1500 U/mL) and 975 mL of milli Q (mQ) water were added (for a total volume of 10 mL) and stirred at 100 rpm, at 37 °C for 2 min in a water bath. A 2 mL aliquot was obtained, centrifuged for 5 min at 8000× *g* and frozen. For the gastric stage, 8 mL of the solution obtained after the oral stage (10 mL of initial volume minus the 2 mL aliquot) was mixed with 6 mL of SGF at 37 °C, 5 μL CaCl_2_ (0.15 mM), 0.16 mL of 1M HCl, 1.28 mL of pepsin from porcine gastric mucosa (25,000 U/mL) and 0.551 μL H_2_O (16 mL total volume), pH was adjusted to 3 with 1 M HCl and stirred at 100 rpm at 37 °C for 2 h in a water bath. A 2 mL aliquot was obtained, centrifuged for 5 min at 8000× *g* and frozen. Then, 7.7 mL of SIF at 37 °C was added to the volume obtained after the gastric phase (14 mL), its pH was adjusted to 7.0 with NaOH 1 M, and 3.5 mL of pancreatin from porcine pancreas (800 U/mL), 1.75 mL of bile salts (160 mM) and 0.91 mL mQ H_2_O were added; pH was verified and adjusted to 7.0 and volume adjusted to 36 mL with SIF if necessary. Samples were stirred at 100 rpm, for 37 °C for 2 h in a water bath. A 2 mL aliquot was obtained, centrifuged for 5 min at 8000× *g* and frozen. These aliquots were used to quantify bioactive compounds as described in previous sections. The aforementioned procedure was performed in triplicate and is graphically described in Figure 1.

A series of additional digestions were prepared, from which no intermediate sampling was performed during the oral, gastric and intestinal stages, in order to allow the full volume to reach the dialysis stage. For this final stage, five sets of triplicate digestions were prepared. Next, 20 of the 40 mL of the final volume of each individual digestion were placed inside a dialysis bag (14 kDa molecular weight cutoff) and submerged in 30 mL of SIF. Each triplicate reaction was individually sampled (2 mL aliquots) at times 0, 30, 60, 90 and 120 min of the dialysis period, on the outside of the dialysis bag. The recovered aliquots were used to identify and quantify the phenolic and carotenoid profiles, as previously described. The bioactive profile of stevia has already been reported elsewhere [15,16,33]. Moreover, the main focus of the present study is guava and mamey pulps; thus, in vitro digestion and subsequent analyses are reported for guava and mamey mixtures only (without stevia).

### 2.15. Statistical Analysis

Results were reported as the mean ± standard deviation. Analysis of variance (ANOVA) was applied to the results obtained from the sensory analysis to determine statistical differences (*p* < 0.05) using Minitab Statistical Software Version 21.0 (State College, PA, USA). Graphs were plotted on SigmaPlot Version 13.0 (SYSTAT Software, Chicago, IL, USA). Design, optimization, graphs and statistical analysis of the beverage were performed with Design–Expert Version 13.0 (Stat-Ease Inc., Minneapolis, MN, USA).

## 3. Results

### 3.1. Total Phenolics and Carotenoids Content

Table 2 shows the bioactive concentration (TPC and TC) and antioxidant capacity (by three different methods) of each of the ingredients used in the formulation, in order to establish a baseline for what the beverages were expected to have. It should be noted that the values for TPC reported here for guava, mamey and stevia, as well as the beverages made from them mentioned throughout the present work, were not obtained with the commonly used Folin–Ciocalteu reagent, but with the Fast Blue BB method. Thus, direct comparison with the existing literature may not be possible, if the Folin–Ciocalteu method was used to obtain it. Although the Folin–Ciocalteu method is the most common method used for the quantification of phenolics, it has been shown to be sensitive to interference from numerous compounds present in fruits (as well as other matrices), while the Fast Blue method is more selective and sensitive without the need for additional extraction and purification steps [34].

It is apparent that the fruits used contributed contrasting types of compounds; for example, guava contains approximately ten times more hydrophilic phenolics than mamey, while mamey contains approximately ten times more hydrophobic carotenoids than guava. Thus, the use of fruits with differing profiles of bioactive compounds contributes to obtaining a richer final matrix. The formulations established by the software (Table 1) were made, and their bioactive compound concentration (TPC and TC) and antioxidant capacity (by three different methods) were quantified, as shown in Table 3. These data show how the combination of different pulp ratios affects the final values of bioactives and antioxidant capacity, and how these variations are more evident on some variables than others. For example, FRAP values varied by approximately three fold when comparing mixtures 3 and 13, while their impact on TEAC is less marked. It has been mentioned that guava extracts are rich in phenolic compounds, which are potent radical scavengers, and may therefore be a good source of natural antioxidants with potential applications in food development [35]. In contrast, mamey’s carotenoid content has been recognized in particular; in this sense, sixty-two carotenoids and carotenoid esters in saponified and non-saponified mamey pulp extracts have been identified, with neoxanthin, cryptocapsin, luteocanthin and capsoneoxanthin being the most representative molecules [36]. It is apparent that aqueous extracts from Stevia leaves contain a high concentration of phenolic compounds (15.50 mg/g) and a high antiradical activity against DPPH and ABTS cation radicals [33].

Regarding the formulated beverages, results of TPC ranged from 248.01 ± 8.21 to 534.34 ± 37.82 mg GAE/100 mL, with mixture 3 having the highest value, according to the formulation of X_1_ = 16.38 g, X_2_ = 20.24 g and X_3_ = 1.37%, and mixture 8 the lowest with X_1_ = 17.77 g, X_2_ = 19.23 g and X_3_ = 1.50%. Others report TPC data for an açaí and lime juice beverage of 1227 mg GAE/100 g, and a “miracle juice mix” with 1795 mg GAE/100 g [37], while a pomegranate and blueberry juice mixture had values of 637 mg GAE/100 mL [20]. A moderate positive correlation was observed between TPC and the amount of guava when it is mixed with other matrices, which can be attributed to its content of phytochemicals, phenolics in particular, but vitamin A, vitamin C and terpenes, among others, could also be contributing [38].

The FBBB method, which quantifies TPC present in a sample, is based on the coupling of phenolic compounds with a diazonium salt, resulting in the formation of azo complexes. Aromatic diazonium ions normally couple with active substrates such as phenolics, in contrast, the commonly used Folin–Ciocalteu method is sensitive to interferences from other reducing compounds, especially with non-phenolic antioxidants and reducing substances (ascorbic acid, glucose, fructose, sulfites) that are common food additives or are naturally present in juices, fruits and vegetables. Amino acids like tyrosine and tryptophan, as well as proteins that contain them, also react with the Folin–Ciocalteu reagent [37].

Several authors have mentioned that the addition of stevia leaves as a beverage sweetener can significantly enhance their TPC and antioxidant capacity [8,16], although this effect was not apparent in our samples; on the contrary, as the percentage of stevia solution increased, TPC and antioxidant capacity decreased. In this sense, interactions between phenolic compounds in the beverage may occur due to the formation of hydrogen bonds between them, leading to a decrease in the ability of hydroxyl groups to neutralize free radicals, and an antagonistic effect [39]. In the same way, this behavior was reported and attributed to the presence of some phenolic acids like gallic, protocatechuic, chlorogenic and vanillic acids [38].

Regarding TC, they ranged from 0.11 ± 0.00 to 0.37 ± 0.01 mg β-carotene/100 mL, which was obtained from mixture 1 (X_1_ = 17.77 g, X_2_ = 18.73 g, X_3_ = 1.5%) and mixture 15 (X_1_ = 12.22 g, X_2_ = 24.78 g, X_3_ = 1%), respectively. Compared to a juice developed from mango, papaya and stevia leaves with 0.67 mg β-carotene/100 mL, it is apparent that our beverage has lower values; this can be attributed to the fact that their sample had two matrices, both of which are rich in these compounds [7]. Also, carotenoid instability and losses are a major concern in maintaining food color, as well as their beneficial effects on health. These losses may be due to enzymatic or non-enzymatic oxidation and geometric isomerization of its polyene chain. Oxidative degradation depends on the availability of oxygen, is stimulated by light, heat, metal ions, enzymes and peroxides, and is inhibited by antioxidants [40,41].

### 3.2. Antioxidant Capacity

An antioxidant is a molecule capable of inhibiting the oxidation of another molecule. Antioxidants break the free radical chain of reactions by donating their own electrons to free radicals, without becoming free radicals themselves. Hydrogen atom transfer (HAT) and electron transfer (ET) mechanisms occur in parallel in most systems, although one may predominate depending on the molecular structure and properties of the antioxidant being studied [42].

Antioxidant capacity by the DPPH method ranged from 156.17 to 257.66 mg TE/100 mL, with mixtures 5 (X_1_ = 14.99 g, X_2_ = 21.50 g, X_3_ = 1.5%) and 11 (X_1_ = 17.77 g, X_2_ = 19.23 g, X_3_ = 1%) having the lowest and highest values, respectively. The TEAC antioxidant capacity ranged from 54.88 ± 1.35 to 92.83 ± 5.24 GAE/100 mL, with mixture 1 (X_1_ = 17.77 g, X_2_ = 18.73 g, X_3_ = 1.5%) having the lowest value and mixture 11 (X_1_ = 17.77 g, X_2_ = 19.23 g, X_3_ = 1%) the highest. In another study, the antioxidant capacities of cranberry juice (measured by DPPH and TEAC methodologies) were reported as 68.5 and 47.1 mg TE/100 mL, respectively [43]. Their samples were subjected to ultrasound and high pressure, as compared to the beverage reported herein, which had higher values measured by those methods, and was not subjected to treatments that could have decreased their antioxidant potential. The higher antioxidant capacity of our beverage may be attributed to the presence of guava, whose bioactives have shown significant ability to scavenge free radicals. In particular, the presence of phenolic compounds like ellagitannins, flavones, flavonols, proanthocyanidins, dihydrochalcones and anthocyanidins, as well as non-flavonoids such as phenolic acid derivatives and stilbenes, among others, has been reported [12].

A study of a stevia beverage in combination with honey and curcumin showed that mixing two matrices with stevia leaves resulted in decreased antioxidant capacity, as measured with the DPPH method. The authors mentioned that decreased antioxidant capacity is commonly related to the presence of certain polyphenolic compounds; for example, combining the stevia beverage with curcumin, a high carotenoid source, decreased its total phenolic content [44]. It is therefore crucial to quantify bioactives in order to document potential synergies/antagonisms that may be taking place.

### 3.3. Model Development and Validation

In the preparation of mixed products, mixture design is a very powerful tool that is commonly used for optimization, since it allows to determine which combinations of factors and levels provide the best responses. Experiments involving this methodology are suitable for the study of products involving more than one ingredient, such that the levels and proportions of the components in the mixture are dependent on each other [9]. Desirability is a widely used mathematical tool for simultaneous optimization since it provides a way to transform the predicted values for each of the variables into one of global convenience.

Once the 16 mixtures were tested, the experimentally obtained values of the dependent variables were entered into the statistical software. Data were fitted into a special cubic model, which considered the effects of the five dependent variables, namely, DPPH (Y_1_), TEAC (Y_2_), FRAP (Y_3_), TPC (Y_4_) and TC (Y_5_), and an optimal mixture was obtained. The ANOVA performed showed a significant result for the model, except for Y_3_. Overall, a close association was found between the experimental values and the predicted ones, indicating that the developed model was satisfactory.

Based on the experimental data shown in Table 3, Table 4 shows the equations that predict how each dependent variable (TPC, TC and antioxidant capacity by three different methods) will respond to any changes to the independent variables (amount of fruit pulp and stevia solution). These were obtained from a special cubic model, by the Design–Expert software. The significant contribution of each coefficient was determined by its *p*-value (*p* < 0.05).

The model’s fitness is generally assessed according to a lack-of-fit test (*p* > 0.05). The *p*-values obtained for DPPH, TEAC, TPC and TC indicated the suitability of the model to accurately predict the variations for most variables, except for FRAP (Y_3_). The quality of the model can be determined based on its coefficients of determination (R^2^), which were 0.72, 0.70, 0.47, 0.73 and 0.75 for DPPH, TEAC, FRAP, TPC and TC, respectively. FRAP did not fit the model according to its *p*-value, R^2^ and lack of fit.

Significant adequacy of the model was confirmed by an R^2^ > 0.7. *p*-values < 0.05 were obtained, except for FRAP (Y_3_), which had a *p*-value = 0.32. This allows us to propose that the models are good descriptors of the experimental data and can be used to estimate the content of TPC, TC and antioxidant capacity by the DPPH and TEAC methods.

In this sense, ref. [9] managed to optimize the nutritional content of a mixture of orange, pineapple and persimmon juices, where a formulation of 33% juice from each fruit had the highest antioxidant capacity and TPC, using a similar methodology to the one reported herein. They obtained R^2^ values between 0.70–0.98 for their variables, and a significant *p* value < 0.05 for their model, thus concluding that said model is adequate to predict their response variables. On the other hand, ref. [45] optimized lycopene content in tomato juice from its fermentation with *Saccharomyces cerevisiae*, concluding that their model was significant, with *p* values = 0.04 and an R^2^ = 0.82. This allows us to suggest that our models are adequate to predict the behavior of the dependent variables.

The prediction models of the response variables were used to determine the mixture of ingredients that guaranteed a maximum concentration of TPC, TC and antioxidant capacity.

#### 3.3.1. Numerical Optimization Computation

In addition to the model validation in terms of the numerical application of the solution variability, an overall desirability function (D) is incorporated and utilized as a metric for multicriteria optimization. For each criterion, fixed values ranging from 0 to 1 are defined, such that the desirability function scale satisfies the following conditions of 0 ≤ d(yi) ≤ 1. If 0 is obtained, it implies that one or more criteria are situated outside their acceptable limit values; if 1 is obtained, it implies that the solutions obtained lie exactly at the acceptable response limits, and the acceptability or rejection conditions depend generally on the objective function, which is defined as the optimization direction through the minimization and maximization of the target through the reference equations [46]. A minimized response indicates that a minimum or lower value is desired. The desirability function for this case is represented in Equation (2).

(2) $$d\left(yi\right)=\left\{\begin{array}{c} 0\\ 0 \end{array}{\left(\frac{U-yi}{U-T}\right)}^{ri}\begin{array}{c} yi<T\\ T\leq yi\leq U\\ yi>U \end{array}\right\}$$

For the second condition, a maximized response indicates that a larger value is derived. The desirability function for this case is represented in Equation (3).

(3) $$d\left(yi\right)=\left\{\begin{array}{c} 0\\ 1 \end{array}{\left(\frac{y-L}{T-L}\right)}^{ri}\begin{array}{c} yi<L\\ L\leq yi\leq T\\ yi>T \end{array}\right\}$$

Finally, for this condition, the target response indicates the best value (response). The desirability function for this case is presented in Equation (4).

(4) dyi=yi<Lyi<UU−yiU−TT≤yi≤Uy−LT−LL≤yi≤T00

where T is the target or actual (experimental) value, yi is the model predicted value of the *i*th response, L is the lower limit or lowest acceptable results or values, ri is the weighted function of the *i*th desirability function, and U is the maximum or highest acceptable results or values. Based on the given conditions presented in the aforementioned equations, a multi-response numerical optimization is executed, through which the optimum mix combination is maximized through the weighted geometric mean of individual desirability function d(yi) from the feasible composite space [47]. Through this optimization process, a model with equal weight is then adopted through composite desirability by using the mathematical equation of the form, where n is the total individual number of responses in Equation (5).

(5) $$D={\left[d(y1)\times d(y2)\times d(y3)...\;\times \;d(yn)\right]}^{1/n}$$

#### 3.3.2. Optimization Overview

Numerical optimization uses the model to search the factor space for the best tradeoffs to achieve multiple goals. The optimization model searches for a combination of factor levels that simultaneously satisfy the criteria placed on each of the responses and factors. The goals that apply to both responses and factors were set to be in the range for the factors and maximize the response, where the lower limit is the lowest acceptable outcome and the upper limit is the desired best result. The statistical software was used to optimize the five dependent variables, which yielded the data shown in Table 5. This table shows the constrained information predefined for each independent variable, as well as the lower and upper limits that were predicted for each dependent variable. It also shows the goal for each variable; for example, the goal for each independent variable was to maintain it within the specified range, while the goal for all dependent variables was to maximize it. Lower and upper weights were similar for all variables, as well as their importance.

From the results of Table 3 and statistical analysis of Table 4 and Table 5, Table 6 shows that the software proposed two optimized formulations, which accomplished the aforementioned goals of maintaining the independent variables within range and maximizing the values of the dependent variables. From the two optimized formulations obtained, the first one was selected due to its higher desirability value. This theoretical formulation represents what is expected to be obtained when experimentally made. In the desirability function computation, the solution with the highest score is preferentially taken as the optimal solution. A desirability criterium score of 0.785 and optimal ratio of 17.77:19.23:1.00 for the fraction of guava, mamey and stevia solution, respectively, were obtained.

### 3.4. Post Analysis and Model Simulation

The post-analysis result presents the confirmation report at the confidence interval of 95%, the descriptive statistical computation of the D-optimal model predicted results and the imposition of constraints.

#### 3.4.1. Point Prediction

Point prediction uses the models’ fit during analysis and the factor settings specified on the factors tool to compute the point predictions and interval estimates. The predicted values are updated as the levels are changed. Similar to Table 6, Table 7 provides more detailed information about the theoretical beverage, including the predicted mean and median for each dependent variable, in addition to its deviation and error and confidence intervals (CI). This information is useful to validate experimental accuracy since all results are expected to be close to these predicted values.

#### 3.4.2. Model Simulation

The model simulation stage is the final phase of the model validation process, where a real-life situation is replicated through what we have modeled to guide users, designers and food developers, among others, on how the developed mixture design will perform. Furthermore, the essence of model simulation is to show that the validation achieved during statistical inference and diagnostics can be achieved in real-life applicability or situations. An ANOVA was used to determine if there was a significant difference between the actual experimental values and the generated simulated mixture design model results. The computations were carried out by substitution of the developed model coefficients to the factor levels for mixture design formulation.

ANOVA results yielded *p*-values of 0.064, 0.384, 0.1, 0.730 and 0.471 for DPPH, TEAC, FRAP, TPC and TC, respectively. Table 8 shows the predicted and experimental values for each independent variable, for the optimized beverage chosen. No statistically significant differences were found between predicted and experimental values, which supports the validity of the model and the accuracy of the experimental procedures.

### 3.5. Phenolic and Carotenoid Profile, and Their Release during In Vitro Digestion

Subsequent experiments regarding the phenolic composition of the digested beverage were carried out only on the optimized one (without stevia solution); results can be seen in Table 9 (marked with an arrow the fraction where the phenolic compound was found) and Table 10. Regarding phenolic compounds, gallic acid was the most abundant, followed by *p*-coumaric acid, gallocatechin gallate, cinnamic acid, quercetin-3-β-d-glucoside and ferulic acid. It has been reported that the main phenolics in mamey fruit are three hydroxybenzoic acids (*p*-hydroxybenzoic, protocatechuic and gallic) and a flavan-3-ol (epicatechin). It has also been reported that *p*-hydroxybenzoic acid is the most abundant compound, with 484 mg/100 g dw, followed by 1.92 mg/100 g dw of gallic acid, 0.78 mg/100 g dw of epicatechin and 2.08 mg/100 g dw of protocatechin [48]. In addition, Ref. [49] reported gallic acid, gallocatechin, catechin, epicatechin, dihydromyricetin, catechin-3-*O*-gallate and myricetin in mamey. Regarding guava, some of the main phenolic compounds identified include catechin, quercetin, gallic acid, epicatechin, luteolin and kaempferol, with 391.93, 122.23, 99.15, 58.43, 51.39 and 38.06 mg/Kg dw, respectively [50].

Other phenolic compounds were identified, namely, *p*-coumaric acid, ferulic acid and chlorogenic acid, but their concentration was below the limit of quantification of our method.

Phenolic compound’s hydrolysis transformation may be due to the acidic environment of the stomach and their poor stability to pH changes, particularly those that are weakly bonded to the food matrix, such as gallic acid, caffeic acid and ferulic acid, among others. This is also influenced by additional interactions of phenolic compounds with the food matrix, as well as their chemical structure. In this sense, the decrease in concentration in pineapple juice of gallic and caffeic acids after in vitro digestion was reported, similarly, ferulic acid in red fruit juice was also reported, probably due to poor stability of these compounds to pH changes [51].

Carotenoid quantification revealed β-carotene and α-carotene as the most abundant species, while cryptoxanthin, lutein and zeaxanthin were also quantified at lower concentrations. Moreover, during in vitro digestion, retinol became detectable only during the different stages of the in vitro digestion, while lutein and zeaxanthin were not detectable. Cryptoxanthin, α-carotene and β-carotene were detected during the oral and gastric phases, but could not be detected during the intestinal phase. Carotenoids like β-carotene, sapotexanthin, cryptocapsin, β-cryptoxanthin, neoxanthin and lutein, have been identified in mamey pulp. In this sense, two important carotenoids found in mamey fruit (β-carotene and β-cryptoxanthin) are provitamin A and, therefore, this fruit has been suggested as an important source of vitamin A in the diet, especially in areas where deficiency is likely to be present [48]. Pro-vitamin A carotenoids such as β-carotene, α-carotene and β-cryptoxanthin provide vitamin A activity in the diet of higher animals like humans. During intestinal digestion, β-carotene is converted to retinal and then reduced to retinol [52]. This can explain the reduction of β-carotene content during the digestion stages and the increase in retinol during the intestinal phase.

### 3.6. Sensory Acceptability

Some of the most important parameters that define the quality of a beverage are its color and overall appearance; taste properties like flavor, mouth persistence and aftertaste; olfactory properties like aroma, odor, orthonasal and retronasal; and tactile properties like mouth feel, body and absence of contaminants (odors and strange flavors) [53].

**Figure 2:**
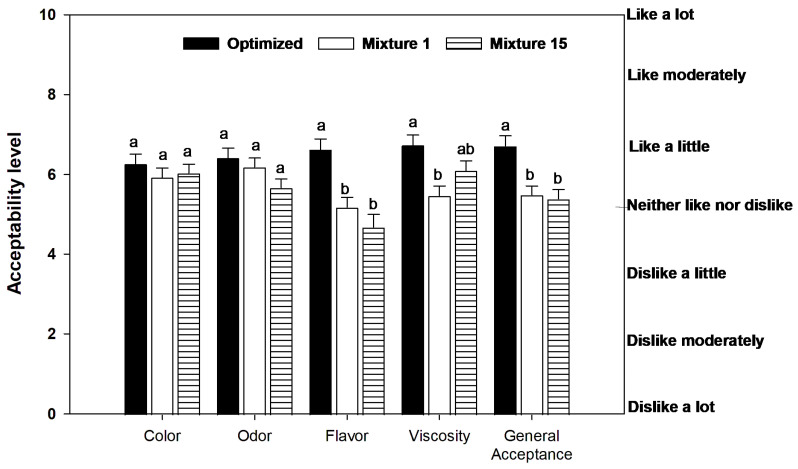
Consumer evaluation (*n* = 110) of the optimized beverage and mixtures 1 and 15. Different letters indicate significant differences (*p* < 0.05).

The consumers who evaluated the beverage (*n* = 110) were 54.54% male and 45.45% female, aged 19–29 (86.4%) and 30–39 (13.6%). They were students, professors and administrative staff from CIAD and the University of Sonora. They were asked to evaluate three formulations (the optimized beverage, and mixtures 1 and 15), and to rate their color, odor, flavor, viscosity and general acceptance. The results of the sensory analysis are shown in Figure 2.

In general, the optimized beverage had scores higher than 6.0 (on a seven-point scale), which corresponded to “like a little” on hedonic terms, specifically, a general acceptability of 6.72. This suggests that all attributes evaluated were well-received and accepted by the consumers. There was no statistically significant difference between the optimized beverage and mixtures 1 and 15 regarding color and odor. In contrast, there were significant differences regarding flavor and general acceptability, where the optimized beverage was better evaluated, suggesting that it had a good sensory balance.

It has been mentioned that the population prefers grit-free, clear, haze-free fruit juice, and clarified guava juice in particular may be more acceptable to the general population [54]. Mamey is considered an exotic fruit in northwest Mexico (where the study was conducted), and the optimized beverage found a good proportion of pulps to be added to produce a well-liked beverage by the local populace. The flavor of mixtures 1 and 15 appeared to be slightly disliked, according to the statistical differences observed. In this context, low acceptability has been reported when evaluating some formulations based on exotic fruits, such as the case of araçá, buriti, cagaita, yellow mombin, mangaba and marolo juices, where all formulations had relatively low scores [55]. The authors of that study attributed these results to the fact that said fruits were not typical of the region where they were evaluated and, therefore, consumers were not accustomed to their flavors.

The initial stages of carotenoid oxidation involve epoxidation and cleavage to apocarotenals, while subsequent fragmentations result in compounds of low molecular masses. Now devoid of color and biological activity attributed to carotenoids, these end-products can give rise to undesirable flavors or off-flavors [41]. According to the comments of sensory evaluation, it can be argued that these processes had not yet occurred to detectable thresholds, and the condition and maturity of mamey pulps were appropriate for the preparation of the beverages reported herein.

It was found that 58 and 64% of male and female consumers, respectively, expressed a positive purchase intention for the optimized beverage. In this sense, it has been reported that female consumers tend to be more conscious about their overall health, which might explain why they tend to value foods that they perceive as healthy while avoiding foods that are high in calories or are otherwise bad for their health. This may explain their increased preference for the optimized beverage, since its ingredients are fruits that can be associated with numerous health benefits, while also being sweetened with stevia [56]. In the same way, it has been reported that men tend to consume more carbonated sugar-rich beverages and fewer fruits and vegetables than women, in addition to other products that can be classified as “unhealthy” [57]. Considering that male consumers reported a high purchase intention, it may be a good option to help avoid traditional sugar-rich products, thereby promoting healthier eating habits.

## 4. Limitations and Impacts

Our work shows promising results for the use of a combined fruit matrix to produce a well-liked beverage, with an optimized bioactive compound concentration; however, there are some limitations that should be mentioned. Most participants were university students, which implies a pre-sampling homogeneity due to them being of similar age, education and interests. Although other older participants were included, they were also from a similar university environment. Mexico’s population is highly varied regarding education, financial status, cultural background and other characteristics; thus, our sampled population only focused on a particular highly defined group. Moreover, children or adolescents were not considered, which is particularly relevant in a country where consumption of sugar-rich carbonated beverages is a main driver of obesity, type 2 diabetes and related conditions from a young age. In order to have a significant impact on such diet-related health issues, good dietary habits should be promoted as early as possible in order to prevent their development instead of treating them.

In contrast to these limitations, we believe that our work can nevertheless have positive impacts, according to the researchers’ interactions with the participants during sensory analysis. For example, some of them expressed that they had not previously consumed or heard of mamey since it is not a staple of northwest Mexico’s diet. Others also commented that they disliked guava, and thus were not regular consumers, but they nevertheless found the combined beverage to be pleasant and said that they would be open to consuming it. These positive observations of a previously unknown or disliked fruit suggested an openness to new and healthy alternatives to incorporate into their diets due to the innovative way in which they were presented to them. We therefore believe that exotic fruits can extend their market to additional geographical locations and be accepted by at least some subsets of the local population; if fresh fruit is not possible to sell due to shelf-life limitations, the development of a healthy product can be an option, either by itself or combined with another fruit, as shown in the present work.

## 5. Conclusions

The present study reports a numerical optimization and in vitro digestion of an exotic fruit-based beverage. The final optimized mixture was 17.77 g of guava pulp, 19.23 g of mamey pulp and a 1% stevia solution, which maximized the content of total phenolics and carotenoids, as well as the antioxidant capacity (DPPH, TEAC and FRAP methods) on the final product. The mathematical models were significant and, therefore, good descriptors of the experimental data for all but one variable (FRAP), while also having good desirability. Some phenolic compounds were identified, with gallic acid as the most abundant one; nevertheless, it was not detected during in vitro digestion. Regarding carotenoids, α-carotene, β-carotene and retinol stood out, with β-carotene being one of the most significant ones due to its pro-vitamin A activity. High concentrations of retinol were also found, suggesting that this beverage could help to achieve the recommended daily intake of this compound. Results of a sensory analysis suggest that the beverage had a good sensory balance, according to its good general acceptance, as well as for specific parameters like color, smell, flavor and viscosity. This was further reflected by a high positive purchase intention by both male and female consumers, which allowed us to propose that it could be well accepted on the market. Further studies are required to determine its postprandial health effects on the consumer.

## Figures and Tables

**Table 1:** Mixture design from guava pulp, mamey pulp and stevia solution.

Mixture	Space Type	Guava Pulp (g)	Mamey Pulp (g)	Stevia Solution (%)
1	Vertex	17.77	18.73	1.50
2	CentEdge	12.22	24.53	1.25
3	AxialCB	16.38	20.24	1.37
4	Vertex	12.22	24.28	1.50
5	CentEdge	14.99	21.50	1.50
6	Center	14.99	21.75	1.25
7	Vertex	17.77	19.23	1.00
8	Vertex	17.77	18.73	1.50
9	Interior	13.60	23.14	1.25
10	CentEdge	14.99	22.00	1.00
11	Vertex	17.77	19.23	1.00
12	Vertex	12.22	24.28	1.50
13	CentEdge	17.77	18.98	1.25
14	CentEdge	14.99	22.00	1.00
15	Vertex	12.22	24.78	1.00
16	Vertex	12.22	24.78	1.00

Limits were established according to [17,18].

**Table 2:** Total phenolic content, total carotenoids and antioxidant capacity of each individual food matrix expressed in wet weight.

Sample	TPC mg GAE/100 g	TC µg β-Carotene/ g	DPPH mg TE/100 g	TEAC mg TE/100 g	FRAP mg TE/100 g
Guava pulp	3632.02 ± 216.55	4.92 ± 0.20	21.98 ± 2.14	68.50 ± 0.66	74.84 ± 2.26
Mamey pulp	316.55 ± 3.59	49.29 ± 1.42	12.35 ± 1.09	46.83 ± 4.45	18.89 ± 1.11
Stevia leaves	540.74 ± 8.98	36.97 ± 0.14	297.63 ± 2.13	102.72 ± 4.40	235.81 ± 7.84

*n* = 3 ± SD.

**Table 3:** Total phenolic content, total carotenoids, and antioxidant capacity by DPPH, TEAC and FRAP of the different formulations obtained from D-optimal mixture design.

Mixture	X_1 (g)_	X_2 (g)_	X_3 (%)_	TPC (mg GAE/100 mL)	TC (mg β-Carotene/100 mL)	DPPH (mg TE/100 mL)	TEAC (mg TE/100 mL)	FRAP (mg TE/100 mL)
1	17.77	18.73	1.50	261.73 ± 15.83	0.11 ± 0.00	186.94 ± 9.93	54.88 ± 1.35	90.47 ± 1.40
2	12.22	24.53	1.25	360.96 ± 11.13	0.13 ± 0.01	188.82 ± 5.09	65.03 ± 1.95	77.60 ± 1.94
3	16.38	20.24	1.37	534.34 ± 37.82	0.17 ± 0.01	248.47 ± 3.20	83.24 ± 3.00	155.33 ± 12.03
4	12.22	24.28	1.50	261.56 ± 14.92	0.14 ± 0.01	213.86 ± 5.48	59.95 ± 0.73	91.10 ± 4.71
5	14.99	21.50	1.50	261.22 ± 10.23	0.12 ± 0.01	156.17 ± 3.24	66.02 ± 2.85	94.75 ± 3.97
6	14.99	21.75	1.25	368.59 ± 13.91	0.15 ± 0.00	241.00 ± 3.69	69.51 ± 3.52	61.16 ± 1.90
7	17.77	19.23	1.00	364.58 ± 17.51	0.25 ± 0.01	253.56 ± 1.17	85.01 ± 6.02	159.31 ± 3.64
8	17.77	18.73	1.50	248.01 ± 8.21	0.14 ± 0.00	165.71 ± 3.36	81.84 ± 5.34	90.80 ± 5.51
9	13.60	23.14	1.25	377.24 ± 16.81	0.16 ± 0.00	242.23 ± 3.83	66.39 ± 1.70	76.61 ± 2.60
10	14.99	22.00	1.00	352.74 ± 13.65	0.17 ± 0.00	247.90 ± 3.79	87.97 ± 3.32	145.79 ± 2.86
11	17.77	19.23	1.00	463.73 ± 9.62	0.14 ± 0.00	257.66 ± 2.10	92.83 ± 5.24	133.915 ± 1.04
12	12.22	24.28	1.50	260.21 ± 5.31	0.15 ± 0.00	157.73 ± 5.91	63.52 ± 5.87	91.51 ± 5.07
13	17.77	18.98	1.25	479.81 ± 15.12	0.14 ± 0.01	253.81 ± 2.17	67.49 ± 8.82	165.53 ± 15.20
14	14.99	22.00	1.00	404.79 ± 32.66	0.25 ± 0.01	252.01 ± 2.99	90.43 ± 4.91	125.52 ± 3.45
15	12.22	24.78	1.00	393.21 ± 20.40	0.37 ± 0.01	241.29 ± 1.79	89.93 ± 4.78	103.13 ± 3.07
16	12.22	24.78	1.00	386.83 ± 20.82	0.36 ± 0.03	243.04 ± 1.80	85.35 ± 6.67	125.90 ± 6.17

*n* = 3 ± SD.

**Table 4:** The fitted special cubic model in terms of coded variables for Y_1_, Y_2_, Y_3_, Y_4_ and Y_5_ responses.

Responses	Third-Order Polynomial Equations	*p*-Value	R^2^	Lack of Fit
DPPH (Y_1_)	Y = 5.56X_1_ + 5.45X_2_ − 66.60X_3_ + 0.1626X_1_X_2_ + 73.88X_1_X_3_ + 74.75X_2_X_3_ − 2.75X_1_X_2_X_3_	0.03	0.72	0.2348
TEAC (Y_2_)	Y = 4.48X_1_ + 4.46X_2_ + 55.47X_3_ + 0.0672X_1_X_2_ − 59.04X_1_X_3_ − 60.31X_2_X_3_ + 4.60X_1_X_2_X_3_	0.04	0.70	0.6803
FRAP (Y_3_)	Y = 33.35 X_1_ + 16.39 X_2_ + 3028.02 X_3_ − 62.26 X_1_X_2_ − 3867.78X_1_X_3_ − 3467.90X_2_X_3_ + 1612.22X_1_X_2_X_3_	0.32	0.47	0.0037
TPC (Y_4_)	Y = 8.41X_1_ + 8.24X_2_ − 135.15X_3_ − 0.42X_1_X_2_ + 149.89X_1_X_3_ + 150.76X_2_X_3_ + 11.37X_1_X_2_X_3_	0.02	0.73	0.0396
TC (Y_5_)	Y = −1.62X_1_ − 1.08X_2_ + 94.39X_3_ − 0.90X_1_X_2_ − 110.21X_1_X_3_ − 116.49X_2_X_3_ + 16.44X_1_X_2_X_3_	0.01	0.75	0.4886

DPPH: 2,2-diphenyl-1-picrylhydrazyl; TEAC: Trolox equivalent antioxidant capacity; FRAP: ferric reducing antioxidant power; TPC: total phenolic content; TC: total carotenoids.

**Table 5:** Optimization criteria definition.

Variable	Goal	Lower Limit	Upper Limit	Lower Weight	Upper Weight	Importance
A: Guava pulp (g)	is in range	12.22	17.77	1	1	3
B: Mamey pulp (g)	is in range	18.73	24.78	1	1	3
C: Stevia solution (%)	is in range	1.0	1.5	1	1	3
DPPH (mg TE/100 mL)	maximize	156.17	257.66	1	1	3
TEAC (mg TE/100 mL)	maximize	54.88	92.83	1	1	3
FRAP (mg TE/100 mL)	maximize	61.16	165.53	1	1	3
TPC (mg GAE/100 mL)	maximize	248.01	534.34	1	1	3
TC (mg β-carotene/100 mL)	maximize	0.11	0.37	1	1	3

Limits were established according to [17,18].

**Table 6:** Optimization solutions.

Number	Guava (g)	Mamey (g)	Stevia (%)	DPPH (mg TE/100 mL)	TEAC (mg TE/100 mL)	FRAP (mg TE/100 mL)	TPC (mg GAE/100 mL)	TC (mg β-Carotene/100 mL)	Desirability	
1	17.77	19.230	1.000	262.157	89.389	171.286	433.043	0.198	0.785	Selected
2	12.220	24.780	1.000	233.653	87.492	114.199	382.257	0.350	0.729	

DPPH: 2,2-diphenyl-1-picrylhydrazyl; TEAC: Trolox equivalent antioxidant capacity; FRAP: ferric reducing antioxidant power; TPC: total phenolic content and TC: total carotenoids.

**Table 7:** Point prediction.

Response	Predicted Mean	Predicted Median	Std Dev	SE Mean	95% CI Low for Mean	95% CI High for Mean
DPPH (mg TE/100 mL)	262.15	260.13	32.77	N/A	216.73	317.09
TEAC (mg TE/100 mL)	89.38	88.75	10.73	N/A	74.75	107.31
FRAP (mg TE/100 mL)	171.28	164.73	48.80	N/A	111.79	262.47
TPC (mg GAE/100 mL)	433.04	420.76	69.31	N/A	339.63	552.14
TC (mg β-carotene/100 mL)	0.19	0.19	0.04	N/A	0.13	0.28

DPPH: 2,2-diphenyl-1-picrylhydrazyl; TEAC: Trolox equivalent antioxidant capacity; FRAP: ferric reducing antioxidant power; TPC: total phenolic content; TC: total carotenoids and N/A: standard error was not generated by the software.

**Table 8:** Predicted and experimental values of optimized mixture.

Variable	DPPH (mg TE/100 mL)	TEAC (mg TE/100 mL)	FRAP (mg TE/100 mL)	TPC (mg GAE/100 mL)	TC (mg β-Carotene/100 mL)
Predicted	262.15 ± 32.77	89.38 ± 10.73	171.28 ± 48.80	433.04 ± 69.31	0.19 ± 0.04
Experimental	213.58 ± 5.06	78.90 ± 4.76	234.03 ± 13.76	418.21 ± 2.74	0.20 ± 0.00

DPPH: 2,2-diphenyl-1-picrylhydrazyl; TEAC: Trolox equivalent antioxidant capacity; FRAP: ferric reducing antioxidant power; TPC: total phenolic content and TC: total carotenoids.

**Table 9:** Phenolic compounds identified and quantified on a guava/mamey (17.77:19.23 g) functional beverage after in vitro digestion.

Phenolic Compound	Retention Time (min)	Free Compound	Alkaline Released	Acid Released	Intestinal Phase In Vitro Digestion	Concentration (μg/mL)
Gallic acid	2.93	✓	✓	-	-	932.50 ± 0.73
Gallocatechin gallate	3.05	✓	-	-	-	4.57 ± 0.05
*p*-coumaric acid	10.27	✓	✓	-	NQ	11.75 ± 0.24
Quercetin 3-β-d-glucoside	13.87	✓	-	-	-	1.74 ± 0.08
Ferulic acid	11.53	-	✓	-	NQ	1.53 ± 0.12
Cinnamic acid	18.96	-	✓	-	-	2.48 ± 0.06
Chlorogenic acid	10.77	-	-	-	NQ	NQ
Vanillic acid	4.20	NQ	-	-	-	NQ
Catechin	4.45	NQ	-	-	-	NQ

✓: identified and quantified; -: not identified; NQ: identified but not quantified. Concentrations correspond to the sum of free compounds and released after acid/alkaline digestions.

**Table 10:** Carotenoids identified and quantified on a combined guava/mamey (17.77:19.23 g) functional beverage before and after in vitro digestion.

Carotenoid	Optimized Beverage	Oral Phase	Gastric Phase	Intestinal Phase
Cryptoxanthin	0.01 ± 0.00	0.99 ± 0.00	0.25 ± 0.00	-
α-carotene	0.36 ± 0.02	1.38 ± 0.00	0.38 ± 0.00	-
β-carotene	0.43 ± 0.00	0.21 ± 0.00	0.03 ± 0.00	-
Retinol	-	8.84 ± 0.00	04.45 ± 0.00	5.76 ± 0.00
Lutein	NQ	-	-	-
Zeaxanthin	NQ	-	-	-

-: not identified; NQ: identified but not quantified; units are μg/100 mL.

## Data Availability

Data is contained within the article.
